# Dental Training

**Published:** 1895-09

**Authors:** 


### Editorial.

### DENTAL TRAINING.

QUESTIONS of dental education are occupying much attention in the journals and dental society meetings, especially at our annual gatherings, and are often treated from a narrow and erroneous point of view. There are those—usually who know little or nothing of practical teaching—who feel that they are annually called upon to assail the dental colleges and their methods of instructing; others calling for difficult entrance examinations. Some would have it so high as to exclude all who have not a classical education, etc. Such examinations many of these advocates would themselves utterly fail in were they put to the test.

The school which requires a rigid entrance examination and has a large faculty, teaching a great variety of subjects,—all of which may be good in their way,—may fail to graduate students who will make successful practitioners. Its students may have what Professor Gross designated as "photographic memories and microscopic brains." They may be store-houses of dental and medical theories, and yet fail utterly as practitioners, and never contribute a single fact to the advancement of their profession. As the *Journal of the American Medical Association* says editorially, upon the subject of professional education, "Many writers use the term medical [or dental] education, as if it were something complete or finished. Diplomas are often considered as evidence of this, and are offered as guarantees of scientific skill."

These are sad delusions; a medical or dental education, we may say, is never finished, and the true aim of our schools is, or should be, to train men to observe and to think for themselves, not to overload them with theories. The mere memorizing of the facts which are seen by the microscope would never make a practical microscopist; and this holds true in any other channel of scientific work. The assumption of truths without personal examination, and the inability and want of training *to* examine independently, is too often the case. The facts must be sought for, examined, and compared.

The dental school that trains its students to be explorers, to study accurately the various phenomena of health and disease in and about the dental organs, inspires them to be ever on the alert for new facts, or new conceptions of old ones, who are never learned but always learning, is the ideal one.

Students should not be allowed to accept the facts presented in the lecture- or clinic-room by their teachers, and found in their text-book, as conclusive, but should be trained to verify these by personal examination and experimentations. Too often have students, who by memorizing a few facts as given by the incumbents of the several chairs during the lecture course, been given their diplomas with the full right to practice, and to be accepted as representatives of the profession. But dental teaching is each year, through the efforts of the Association of Dental Faculties, being placed upon a more uniform and broader scale. More time is given to the several laboratories and class-rooms, where the instructions are more of an individual nature, and where the student is taught and encouraged in using his powers of observation and reason, and, in making personal experiments, to be accurate and true. Manual training is an important factor in dental education, and those who were fortunate enough to attend the meetings of the National School of Dental Technics, recently held at Asbury Park, must have been impressed with that fact. Dr. E. C. Kirk, an acute observer, in commenting upon this point, says, "The application of the laboratory method in dental education, the introduction of the technic method in our schools, by bringing the instruction in operative and mechanical dentistry and therapeutics into line with the laboratory method as utilized in the departments of pathology, chemistry, and histology, is a most important step towards cultivating a scientific habit of mind, and a desire for original research among the dental students of to-day, which must tend to elevate our standards and ideals, and react favorably upon future methods of practice."

Now that dental teaching has reached a broader and more uniform basis, our schools should require something more in the way of examinations than recitations and the memorizing of facts. The mere gathering and storing of dental knowledge can never make a successful dentist. While no college can educate a man in the true sense, yet they can prepare him to use his powers of observation and reason; and when a student realizes his limitations and the personal equation of error that is liable to complicate his observations, he becomes a scientist in the highest meaning of that word. Of course all of us cannot be scientific investigators in the broad sense, but, as has been observed by others, it is, after all, a matter of degree, for every one is, or should be, capable of observation, and able to interpret and report such observation.

While many of our colleges are excellent, they have not, as a rule, introduced this matter of personal observation and the recording of same in their curriculum. We have the medical journal referred to above as authority, that an English medical school has recently adopted a plan requiring all senior students to spend a good portion of the last year in observing and writing up cases, the notes of which are corrected by the teachers. In this way the senses and reason are trained to observe and compare the relation of facts,—a move, we think, worthy of emulation. G. W. W.

